# SPIDIA-RNA: Second External Quality Assessment for the Pre-Analytical Phase of Blood Samples Used for RNA Based Analyses

**DOI:** 10.1371/journal.pone.0112293

**Published:** 2014-11-10

**Authors:** Francesca Malentacchi, Mario Pazzagli, Lisa Simi, Claudio Orlando, Ralf Wyrich, Kalle Günther, Paolo Verderio, Sara Pizzamiglio, Chiara Maura Ciniselli, Hui Zhang, Vlasta Korenková, Lynne Rainen, Tzachi Bar, Mikael Kubista, Stefania Gelmini

**Affiliations:** 1 Department of Biomedical Experimental and Clinical Sciences, University of Florence, Florence, Italy; 2 Qiagen GmbH, Hilden, Germany; 3 Unit of Medical Statistics, Biometry and Bioinformatics, Fondazione Istituto di Ricovero e Cura a Carattere Scientifico (IRCCS) Istituto Nazionale dei Tumori, Milan, Italy; 4 DiaGenic ASA, Oslo, Norway; 5 Institute of Biotechnology, Academy of Sciences of the Czech Republic, Prague, Czech Republic; 6 Becton Dickinson (BD), Franklin Lakes, New Jersey, United States of America; 7 Labonnet Ltd, St. Ramat-Hasharon, Israel; 8 TATAA Biocenter AB, Gothenburg, Sweden; The University of Hong Kong, Hong Kong

## Abstract

One purpose of the EC funded project, SPIDIA, is to develop evidence-based quality guidelines for the pre-analytical handling of blood samples for RNA molecular testing. To this end, two pan-European External Quality Assessments (EQAs) were implemented. Here we report the results of the second SPIDIA-RNA EQA. This second study included modifications in the protocol related to the blood collection process, the shipping conditions and pre-analytical specimen handling for participants. Participating laboratories received two identical proficiency blood specimens collected in tubes with or without an RNA stabilizer. For pre-defined specimen storage times and temperatures, laboratories were asked to perform RNA extraction from whole blood according to their usual procedure and to return extracted RNA to the SPIDIA facility for further analysis. These RNA samples were evaluated for purity, yield, integrity, stability, presence of interfering substances, and gene expression levels for the validated markers of RNA stability: FOS, IL1B, IL8, GAPDH, FOSB and TNFRSF10c. Analysis of the gene expression results of FOS, IL8, FOSB, and TNFRSF10c, however, indicated that the levels of these transcripts were significantly affected by blood collection tube type and storage temperature. These results demonstrated that only blood collection tubes containing a cellular RNA stabilizer allowed reliable gene expression analysis within 48 h from blood collection for all the genes investigated. The results of these two EQAs have been proposed for use in the development of a Technical Specification by the European Committee for Standardization.

## Introduction

SPIDIA (Standardization and Improvement of Generic Pre-analytical Tools and Procedures for In Vitro Diagnostics; www.spidia.eu) is a European Commission funded, four-year, integrated project aimed at the standardization and improvement of pre-analytical procedures for *in vitro* diagnostics. Project objectives are accomplished by using evidence-based, quality assurance schemes derived from external quality assessments (EQAs) and validated technologies for the collection, transport and processing of blood samples for *in vitro* diagnostic testing of genomic DNA, cell-free (plasma) DNA, and intracellular RNA [1], [2].

As we noted in our previous publication of results of the first SPIDIA EQA of intracellular RNA [1], the inherent instability of RNA makes planning a well-controlled, external evaluation of this analyte in blood a considerable challenge. While results of the first EQA demonstrated an association between gene expression levels and RNA integrity number (RIN), the results did not indicate significant differences in the expression levels of the investigated genes as a function of storage time, temperature, or whether or not the blood collection tube contained an RNA stabilizer. The first EQA was conducted using pooled blood specimens from different donors collected in citrate phosphate dextrose adenine (CPDA) anti-coagulant. Pooled blood was aliquoted into proficiency specimens and shipped to participating laboratories under uncontrolled shipping conditions. These factors may have caused *ex vivo* changes in expression of investigated genes before RNA analysis. Taking into account some of the problems encountered with this first study, we first investigated the effect on gene expression of blood pooling, and we designed a second, expanded EQA with some modifications related to (i) the blood collection process, (ii) the shipping conditions and (iii) the pre-analytical specimen handling protocol for participating laboratories. Here we report the results of the second SPIDIA-RNA EQA.

Since most blood specimens are collected in EDTA tubes, blood collection for the second study was performed using bags prefilled with an EDTA solution such that the final molar concentration approximated that of EDTA tubes. This step was taken to obtain a large volume of whole blood which closely resembled in composition whole blood specimens received in clinical laboratories, i.e. EDTA whole blood. Because blood from a single donor was not of sufficient volume to provide proficiency specimens to all study participants, two blood donors were enrolled, blood from each donor was aliquoted into T_0_ control and proficiency specimens, the resultant specimens were identified as to donor source, and the results segregated accordingly. The participating laboratories were therefore randomized into two groups, each group receiving proficiency specimens associated with one donor. To maintain constant temperature during sample shipment, we adopted dedicated shipping containers that maintained an internal temperature of 2°C to 8°C for 48 h.

The protocol for participants for the second EQA was virtually the same as for the first EQA study. Briefly, two proficiency specimens, both either with or without an RNA stabilizing additive, were sent to participating laboratories according to whether or not they wished to receive tubes containing stabilizer. Participants were asked to extract the RNA from whole blood sample from one tube immediately after receipt by the laboratory and from the second tube 24 h later after storage at either ambient or refrigerated temperature. Storage temperature was assigned randomly. The participants were instructed to extract the RNA using their routine laboratory procedure and send the purified RNA samples back to the SPIDIA facility (Prof. M. Pazzagli, Clinical Biochemistry Laboratory, University of Florence, ITALY) for analysis.

The quality and quantity of RNA in the returned samples were evaluated by means of the same methodology used in the first SPIDIA-RNA EQA. These methods included the spectrophotometric measurement of total RNA yield and purity, RIN score as measured by the Agilent Bioanalyzer [3], expression levels of the genes FOS, IL1B, IL8, and GAPDH [4], and detection of qPCR inhibition [5]. In addition, the expression levels of two new biomarkers, FOSB and TNFRSF10c, developed and validated within the SPIDIA project, as indicators of *ex vivo* gene expression changes in stored EDTA blood, were also included in order to improve the evaluation of highly labile RNA targets [5], [6]–[8].

The results of these two SPIDIA RNA EQA studies have been compiled and will be used by the European Committee for Standardization (CEN) to propose an evidence-based Technical Specification for pre-analytical handling of blood for RNA-based *in vitro* diagnostics.

## Materials and Methods

### Effect of blood specimen pooling on gene expression

#### Blood collection

Blood from 18 healthy, consented subjects was collected into five Vacutainer K_2_EDTA Tubes (BD, Franklin Lakes, NJ), after ethical approval of the Ärztekammer Nordrhein (German). The healthy subjects signed an informed consent. We made six specimen pools, each pool containing one tube from each of three randomly selected subjects. From each pool as well as from one of the remaining EDTA tubes from each donor, a 2.5 mL aliquot was transferred into PAXgene Blood RNA Tubes (PAXgene) (PreAnalytiX, Hombrechtikon), incubated for 6 h at room temperature, and then frozen at −20°C. The remaining three tubes from the individual subjects, as well as the six sample pools were incubated at room temperature for up to 3 days. After one, 2, and 3 days, a 2.5 mL aliquot of blood from each sample was transferred into PAXgene tubes to stabilize the transcript profile, incubated for 6 h at room temperature, and then frozen at −20°C. At the end of the time course, RNA from all specimens stored in PAXgene tubes was extracted according to the PAXgene Blood RNA Kit Handbook Version 2 and analyzed for individual transcript levels.

#### PCR Analysis

Reverse Transcription quantitative PCR (RT-qPCR) was performed using 3 µL of the RNA eluate. The one-step qRT-PCR reactions were performed as duplex qPCR (FOS/18SrRNA and IL1B/18SrRNA) for 40 cycles on a TaqMan 7700 cycler (ABI) using the QuantiTect Probe RT-PCR Kit (QIAGEN, Germany) and specific primers. Relative transcript levels of FOS and IL1B gene transcripts were determined by duplex RT-qPCR, using 18S rRNA as an internal standard and ΔΔCq calculation.

#### Statistical Analysis

A generalized linear modelling [9] approach was implemented on the qPCR data by considering a model including both factors together with their first order interaction term. This model was implemented by considering the log_2_(RQ) values as dependent variables where RQ = 2^−ΔΔCq^ and −ΔΔCq = [(Cq_gene_−Cq_18S_)_TimeX_−(Cq_gene_−Cq_18S_)_Time0_. Statistical analysis was performed using SAS software v. 9.2 (SAS Institute Inc. Cary, NC).

### Second SPIDIA-RNA EQA

#### Enrollment of Applicants

The announcement of the second SPIDIA-RNA EQA was published on the EFLM web site (www.efcclm.org) which listed the protocols, the application form, and a participant questionnaire. Laboratories applying for participation were asked to describe the type of blood collection tube they usually use for RNA-based analyses: tubes without an RNA stabilizer (e.g. EDTA Tube, EDTA) or with an RNA stabilizer (e.g. the PAXgene Blood RNA Tube, PAXgene).

Details on the content of these web pages are reported as Supporting Information. These include the protocols describing the procedures for blood storage and RNA extraction (Protocol S1, Protocol S2 and, Protocol S3), and the Results Form on which to record the data (Protocol S4, Protocol S5). Three different protocols and Results Forms were finalized depending on the type of blood collection tube used as specified by the applicant. All participants were informed in advance of the shipping date of the samples.

#### Proficiency specimen preparation and shipment

**Figure 1:**
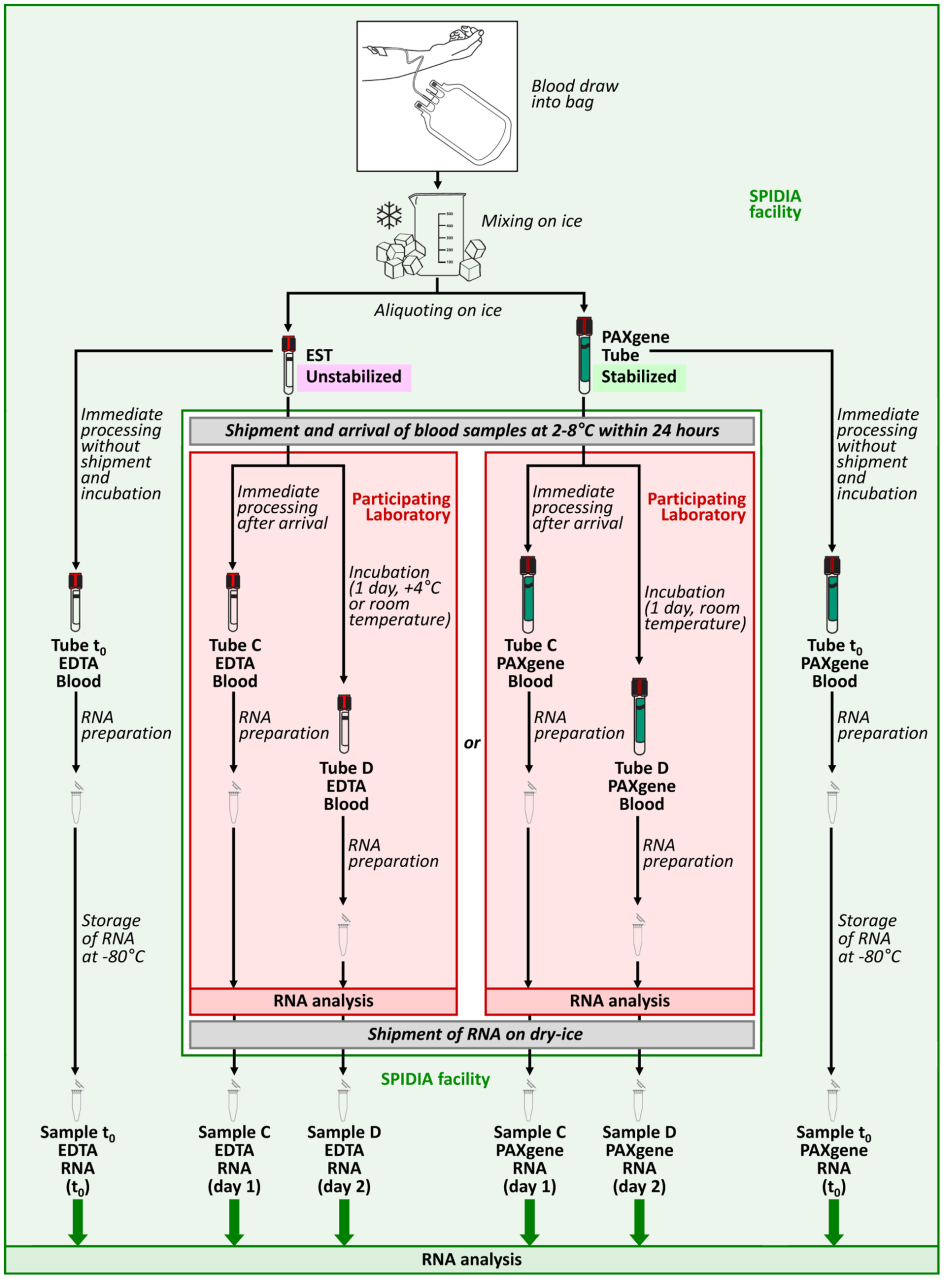
Schematic presentation of the workflow of the second SPIDIA-RNA EQA. Blood was drawn from two donors (D1, D2) into separate EDTA containing bags. EST = Evacuated Secondary Tube, that does not contain any chemical formulation. T_0_ = Blood processed without storage, immediately after blood collection. day 1, day 2 = Time period between blood collection and RNA preparation.

Blood was collected from two consented, adult donors (Donor1, “D1” and Donor2, “D2”) after approval by Institutional Committee of Azienda Ospedaliero-Universitaria Careggi (Florence, Italy). The donors signed a written informed consent. In order to make enough proficiency specimens for all participating laboratories, venous whole blood (approximately 450 mL) was collected from each donor into a blood collection bag (MacoPharma) prefilled with K_3_EDTA (1.79 mg/mL) kindly supplied by BD, Plymouth, UK. Blood from each donor was transferred into a separate, sterilized flask, mixed under gently stirring conditions while cooled on ice, and immediately aliquoted into BD Vacutainer Evacuated Secondary Tubes (ESTs) (BD) (3 mL/tube) and PAXgene tubes (2.5 mL/tube). RNA was isolated immediately from replicate proficiency specimens from each donor and designated as time zero control (T_0_) for gene expression stability studies (Fig. 1). RNA from T_0_ PAXgene Blood RNA tube specimen was extracted by PAXgene Blood RNA Kit, 2.5mL blood from T_0_ EDTA tube was transferred immediately after blood collection in PAXgene Blood RNA tube and extracted by PAXgene Blood RNA Kit. Depending upon the request specified in the application form, each participating laboratory received two proficiency specimens, “Tube C” and “Tube D,” either in ESTs (EDTA whole blood) or in PAXgene tubes. The participating laboratories were randomly allocated into two groups: one group received two specimens from Donor1 and the other group received two specimens from Donor2. PAXgene tubes were incubated at room temperature for 2 h prior to packaging according to manufacturer’s instructions. Aliquoted proficiency specimens were stored at 4°C prior to packaging and shipment, and boxes containing cooled gel packs to maintain 2–8 °C for 48 h were shipped by an international courier on the same day of blood collection.

#### Instructions for the Participants

The laboratories were asked to store the blood at either room temperature (RT) or 4°C and extract the RNA at specified times after receipt. Participants were asked to extract RNA from Tube C (RNA C) immediately upon arrival of the tubes and from Tube D (RNA D) 24 h after Tube C. PAXgene Tube D was stored at RT while EDTA Tube D was stored either at RT or at 4°C according to a randomized scheme. Tube D was therefore used only to investigate the effect of the storage time and temperature on the quality of extracted RNA and not for the proficiency evaluation. The two extracted RNA samples from Tubes C and D (RNA C and RNA D) were analyzed spectrophotometrically by the participating laboratory for concentration and purity (A_260_/A_280_), and both purified RNAs were shipped on dry ice to the SPIDIA facility for further analysis.

#### Data reporting from participants

The participants used the on-line Results Form (Protocol S4 and Protocol S5) to report detailed information of the procedure used during the RNA extraction phase. This information included the date of sample arrival, the temperature and duration of blood sample storage, RNA extraction protocol used, the spectrophotometric evaluation, and temperature and duration of storage of the extracted RNA prior to shipping.

#### Extracted RNA shipment and storage conditions

After RNA extraction, the participants shipped the two RNA samples, RNA C and RNA D, on dry ice back to the SPIDIA facility where the extracted RNA samples were stored at −80°C until analysis.

#### RNA quality parameters

The RNA quality parameters tested at the SPIDIA facility included UV spectrophotometric analysis of RNA purity and yield as determined by the participants and the RIN score as determined by an Agilent Bioanalyzer 2100 (Agilent Technologies) for an overall evaluation of RNA integrity. Further testing of RNA integrity and quality included an RT-qPCR measurement of the expression of FOS, IL1B, IL8 and GAPDH transcripts and analysis of the RT-qPCR kinetics for the detection of the presence of RT-qPCR inhibition. Details on the reagents and methods used for these analyses are reported elsewhere [1], [5], [10].

In particular, primers and probes for GAPDH (Pre-Developed TaqMan Assay Reagents, P.N. 4326317E), IL1B, IL8 and FOS (TaqMan Gene Expression Assay; Hs00174097_m1, Hs99999034_m1, and Hs00170630_m1, respectively) were from Life Technologies. Total RNA (400 ng) was reverse transcribed using a TaqMan Reverse Transcription Reagents kit (Life Technologies). Reverse transcription was performed in a final volume of 80 µL containing 500 mM KCl, 0.1 mM EDTA, 100 mM Tris·HCl (pH 8.3), 5.5 mM MgCl_2_, 500 µM of each dNTP, 2.5 µM random hexamers, 0.4 U/µL RNase inhibitor, and 1.25 U/µL Multiscribe Reverse Transcriptase. The reverse transcription reaction was performed at 25°C for 10 min, 48°C for 30 min, and 95°C for 3 min. Gene expression was measured by qPCR. For each sample 12.5 ng of cDNA was added to 10 µL of PCR mix containing a primer set and 1× Universal PCR Master Mix (Life Technologies). The samples were then subjected to 40 cycles of amplification at 95°C for 15 s and 60°C for 60 s in the ABI PRISM 7900 Sequence Detector (Life Technologies). The amount of each target gene was evaluated against a standard curve. Each standard was obtained by cloning a cDNA fragment of the specific gene (FOS, GAPDH, IL1B, and IL8) into the plasmid pCR2.1-TOPO (Life Technologies). Each standard curve was generated by plotting the mean Cq of the technical replicates versus the logarithm of the known starting concentration [16]. Samples and standards were measured in qPCR triplicates. The gene expression results are reported as log_10_ (copies/µg total RNA).

In addition, two previously validated biomarkers identified within the SPIDIA project which indicated *ex vivo* gene expression changes in stored blood were used to determine the extent of gene transcription instability in stabilized and unstabilized blood specimens (manuscript submitted for publication). These transcripts, one of which is up-regulated (FOSB) and the other down-regulated (TNFRSF10c) in EDTA blood tubes, were quantified in both RNA C and RNA D samples by qPCR relative quantification against T_0_ controls using PPIB and GUSB genes as reference genes. For the qPCR analysis of these four biomarkers, 2 µL of cDNA were added in a total volume 20 µL containing a Quantitect probe PCR master mix (Qiagen) 1x, 100 nM TaqMan probe, 400 nM forward and reverse primers, and water and incubated for 95°C for 15 min and 50 cycles of 95°C for 15 s and 61°C for 90 s each.

### Statistical analysis and results interpretation

#### Evaluation of laboratory proficiency

The evaluation of the laboratory proficiency was carried out by applying the same approach previously described [1]. The aims of the applied statistical procedure were to detect outlier results and/or identify laboratories with issues related to pre-analytical handling of specimens by calculating robust control limits (one or two sided) and comparing lab results to these limits. These consisted of an Action Limit (AL) and a Warning Limit (WL) [11], [12]. According to these limits, the proficiency of each participant was classified as follows:

- Out of control: the value exceed the upper or lower AL or the value was below the one-sided AL.
- Warning: the value was between the upper AL and WL or between the lower AL and WL, or between the one-sided WL and AL.
- In control: the value was between the lower and the upper WL or exceed the one-sided WL.

The analysis and interpretation of the RT-qPCR kinetics were performed as previously described [1], [5].

#### Evaluation of the FOSB and TNFRSF10c Biomarkers

The expression level of these biomarkers was evaluated as relative to housekeeping gene transcripts by comparative Cq method [13] as follows: RQ = 2^−ΔΔCq^ where: −ΔΔCq = [(Cq_biomarker_ – Cq_meanref_) _Time x_–(Cq_biomarker_–Cq_meanref_)_Time 0_ where “meanref” is the mean of the Cq values of the two housekeeping genes, and “T_0_” designates RNA extracted immediately after blood collection at the SPIDIA facility. The evaluation of gene expression was performed within each donor using the corresponding T_0_ values.

#### Influence of blood collection tube type and/or storage temperature on gene expression

The relationship between blood collection tube type alone or in combination with storage temperature and the gene expression levels of the four selected genes was investigated by using a non-parametric approach (Kruskal-Wallis Test). The comparisons were performed by considering the T_0_-adjusted scale of each variable (across-subjects analysis). To account for multiple comparisons, a Bonferroni correction p-value was computed.

All statistical analysis was performed using SAS software v. 9.2 (SAS Institute).

## Results

### Effect of blood pooling on gene expression of FOS and IL1B

**Figure 2:**
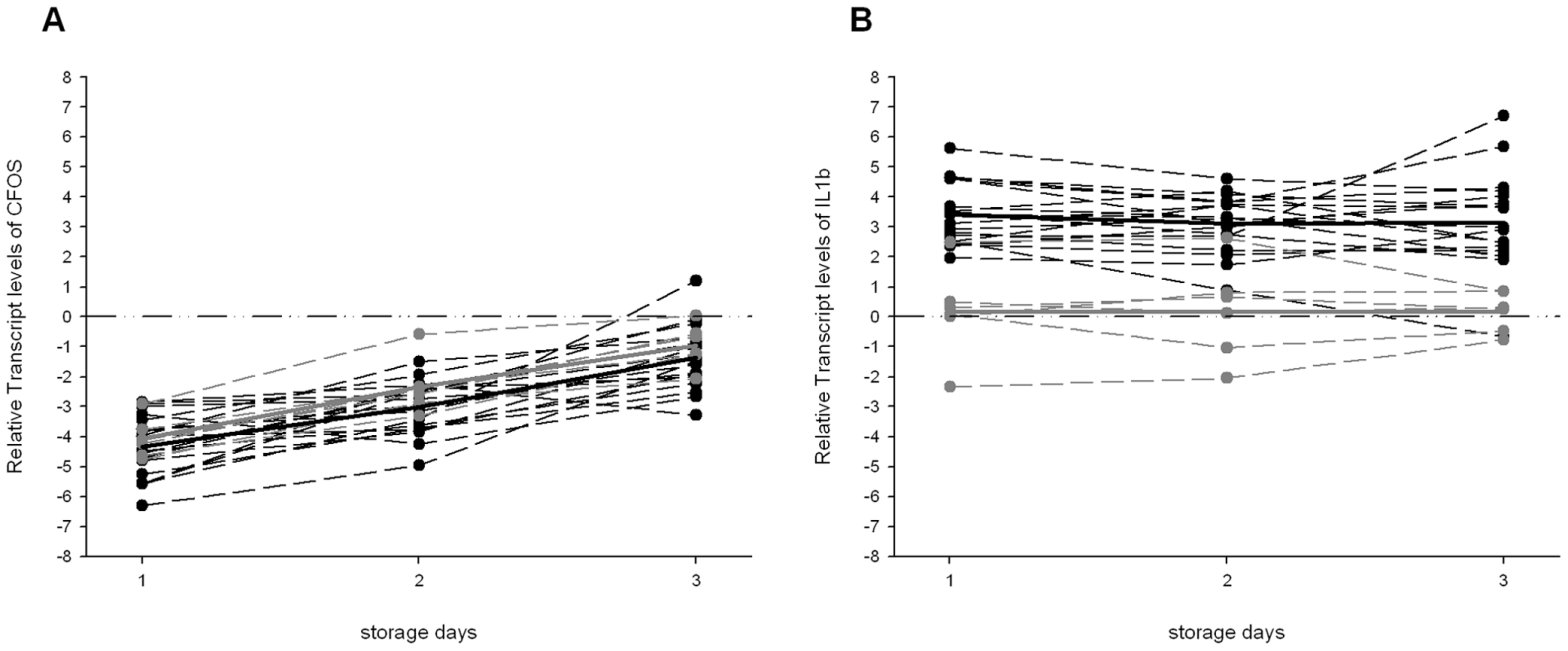
Relative Transcript levels of FOS and IL1B in individual and pools samples. Overall distribution of FOS (A) and IL1B (B) according to time storage. Each dot represents the expression levels of each individual (black) or pool (gray) samples for each time; the dashed lines indicate the time-trend for each sample. The continuous lines indicate the overall trend for individual (black) and pool (gray) samples. The horizontal dot-dashed line indicates the expected value of T_0_.

Expression levels of FOS significantly changed over time (p-value: <0.01) but were not significantly affected by the blood pooling (p-value: 0.09) (Fig. 2A). IL1B showed a statistically significant difference in expression between pooled and not-pooled blood (p-value: <0.01) whereas storage time was not a statistically significant factor (p-value: 0.93) (Fig. 2B).

### Second SPIDIA-RNA EQA

#### Applicant recruitment and questionnaire information

One hundred twenty-two applications (approximately 50% were accredited laboratories) were received from 21 different European countries, and 119 laboratories confirmed their participation in the second SPIDIA-RNA EQA (Fig. S1A). A description of the structure of the participating laboratories is reported in Figure S1B. The most frequently used analytical applications requiring purified RNA are shown in Figure S1C.

At deadline, 109 laboratories (92%) returned extracted RNA to the SPIDIA facility. Eighty (80) of the 109 laboratories had received blood specimens in EST tubes (41 from Donor1 and 39 from Donor2) and the remaining 29 laboratories in PAXgene tubes (15 from Donor1 and 14 from Donor2).

Analysis of the Questionnaire (n = 92 labs) revealed that 66% of the laboratories typically collect blood in EDTA tubes, 21% in PAXgene tubes, and the remaining 13% in other blood collection tubes. The blood volume normally collected by the participating laboratories, ranged from 1 to >10 mL, and most laboratories perform RNA extraction within 12 h post-phlebotomy. Participants indicated that the extracted RNA is mainly used for reverse transcription and subsequent qPCR. These data as well as additional information describing the current methods for RNA extraction and evaluation of RNA concentration are summarized in Table S1. Analysis of the Result Forms revealed that only 42/109 (39%) of the participants used the DNase treatment during RNA extraction, even if it is well known that DNA contamination during RNA purification can lead to non-specific amplification and aberrant results in reverse transcription quantitative PCR [14].

#### Report for the participants

At the SPIDIA facility, the RNA samples sent from the SPIDIA participants were analyzed as described in Materials and Methods and the results evaluated using the statistical approach described above to produce an individual report for each participating laboratory. In each report, the distribution of all the data for each quality parameter was graphically displayed in a box-plot, which included the AL and the WL together with a red dot indicating the individual value of the particular laboratory. A box under each graph indicated the classification of the laboratory’s proficiency for each specific parameter. Appendix S1 shows an example of a report for Donor1.

#### Spectrophotometric data

Tables A.1 and A.2 in Appendix S1 summarize the spectrophotometric measurement results provided by the participants and by the SPIDIA facility along with some details concerning times, methods, and reagents. Sections A.3 and A.4 (Appendix A) depict box-plots of the distributions of RNA purity and yield for RNA C reported by the participants and measured by the SPIDIA facility.

As reported in section A.3, a similar purity distribution within each donor was observed by using values reported by participating laboratories (D1 median = 1.98, IQR = 0.23; D2 median = 2.02, IQR = 0.21) and the SPIDIA values (D1 median = 1.98, IQR = 0.24; D2 median = 1.95, IQR = 0.17) The same findings were observed for total RNA yield (ng/µL blood, section A.4) with a similar, within-donor distribution for both the lab values (D1 median = 2.34 ng/mL, IQR = 2.21; D2 median = 2.22 ng/mL, IQR = 2.15) and the SPIDIA values (D1 median = 1.94 ng/mL, IQR = 2.00; D2 median = 2.01 ng/mL, IQR = 1.85).

#### RIN Scores

Section B.1 (Appendix S1) reports the distributions of RIN scores obtained from RNA C, and section B.2 (Appendix S1) shows the corresponding electropherogram. The median value was similar in the two Donors (D1 median = 8.60, IQR = 2.10; D2 median = 8.15, IQR = 2.10). The WL indicated that the proficiency of a laboratory could be classified as “in control” when the RIN score was greater than 6.90 for Donor1 and 6.62 for Donor2.

#### Gene expression profile

The distributions of the gene expression [log_10_ (copies/µg total RNA)] of the four genes tested are graphically represented in section C.1 (Appendix S1). For both Donors, IL1B showed the narrowest resultant distribution and the lowest variability in comparison to the other genes.

#### qPCR Kinetics analysis

In section D.1 (Appendix S1) we reported the distribution of the Kinetics Distance (KD) obtained by the analysis of the qPCR kinetics data by the Kineret software procedure described by Tichopad, *et al.* [5]. The two lines depicted in the figures correspond to the theoretical limits used to detect strong (>9.21) and weak (5.99−9.21) outliers. For all transcripts, the median value is below the defined thresholds. Of note was that results for GAPDH were available for only 22/56 (39%) and 22/53 (42%) for Donor1 and Donor2 respectively, and therefore this parameter was not considered in the evaluation of the overall performance of the participating laboratories.

#### Summary of the lab proficiency evaluation

Table E in Appendix S1 shows the proficiency of the laboratory for RNA quality parameters evaluated in this study. The table depicted the results with three colors: green indicating “in control”, yellow indicating “warning” or “weak outlier”, and red indicating “out of control” or “strong outlier.” Missing values were designated “missing” in the summary table with an explanation in the “comments” column. All data were visually summarized as a “radar” graph with proficiency level symbolized by a colored square (same colors as in the Summary Table, Table E in Appendix S1). The distance between the colored square and the center of the graph indicates the level of proficiency (the further away from the center, the worse the proficiency).

#### Effects of blood collection tube and storage conditions on FOSB and TNFRSF10c Biomarkers

In section F (Appendix S1) we reported the distributions of the relative quantification of the up- and down-regulated FOSB and TNFRS10c biomarkers with respect to the blood collection tube (Tube C and Tube D) and relative to both blood collection tube and storage temperature (Tube D). In the figures, the horizontal line indicates a log_2_(RQ) = 0 corresponding to the T_0_ value that is expected in the absence of up- or down-regulation.

For both donors, the variations from T_0_ values of FOSB and TNFRSF10c transcripts from blood collected in PAXgene tubes were close to zero even 48 h post-phlebotomy. Messenger RNA species from EDTA tubes, however, showed time- and temperature-dependent expression levels. In particular, at 24 h post-phlebotomy (Appendix S1, section F1 and F2, Tube C), we observed an induction of FOSB expression in comparison to the T_0_ value. Transcript copy number further increased 48 h after collection, especially if the blood samples were stored at RT (Kruskal-Wallis p-value <0.01) (Appendix S1, Section F1, Tube D). The same findings in the opposite direction were observed for the down-regulated biomarker, TNFRSF10c (Kruskal-Wallis p-value <0.01) (Appendix S1, section F2, Tube D).

#### Overall proficiency of the participating laboratories

On the basis of the RNA quality parameters measured in this second SPIDIA-RNA EQA, 45% (D1) and 42% (D2) laboratories were within non-critical proficiency limits (all parameters classified as “in control” or “warning”) whereas 29% (D1) and 30% (D2) laboratories presented one “out of control” rating and/or one or more “missing data” responses. The remaining participating laboratories (D1∶27%, D2∶28%) presented two or more “out of control,” with or without “missing data” quality parameters (Table 1).

**Table 1:** Classification of the proficiency of the laboratories.

	Donor1		Donor2	
Categories	n	%	n	%
all “in control” or “warning” a	25	45	22	42
one “out of control” and/or one or more “missing”^b^	16	29	16	30
two or more “out of control” with or without missing^c^	15	27	15	28
Total of participants laboratory	56	100	53	100

a **all “in control” or “warning”**: labs with all parameters in control or warning, without missing;

b **one “out of control” and/or one or more “missing”**: labs with only one out of control (D1: n = 16, D2: n = 13); labs with only one missing (D1: n = 0, D2: n = 1) or only more than one missing (D1: n = 0, D2: n = 1); labs with one out of control and one missing (D1: n = 0, D2: n = 1); labs with one out of control and more than one missing (D1: n = 0, D2: n = 0);

c **two or more “out of control” with or without “missing**”: labs with two out of control with at least one missing (D1: n = 2, D2: n = 3) or without missing (D1: n = 5, D2: n = 6); labs with more than two out of control with at least one missing (D1: n = 3, D2: n = 3) or without missing (D1: n = 5, D2: n = 3).

An overall increase in the quality of lab proficiency from the first to the second SPIDIA-RNA EQA was observed with the percentage of laboratories with “good” proficiency ratings increasing from 26% in the first EQA to 43% (by considering both donors) in the second EQA.

### Pre-analytical factors and Gene Expression

**Figure 3:**
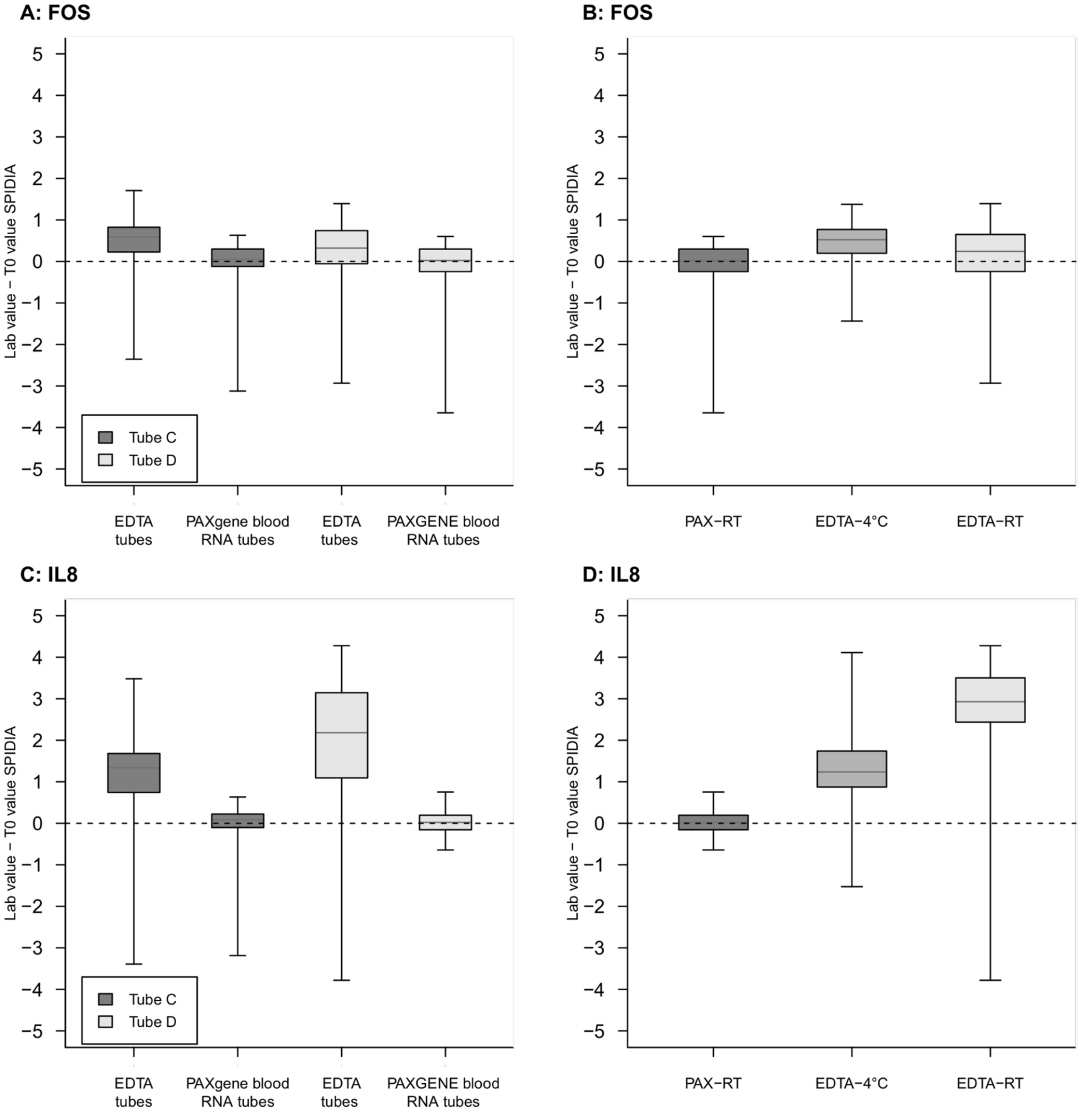
Blood collection tube and/or storage temperature and Gene Expression. Overall distribution of FOS and IL8 according to blood collection tube (3A and 3C, respectively) and to storage temperature/collection tube in RNA D (3B and 3D, respectively). The box horizontal sides identify the 25th and 75th centile, the horizontal line inside the box the median, the two whiskers correspond to minimum and maximum, and the dashed line indicates the T_0_ value zero.

Figure 3A and 3C show the distributions of the gene expression analysis of FOS and IL8 with respect blood collection tube type in RNA C and RNA D. Tube D was used to investigate the effect of storage time, storage temperature and tube type on the quality of extracted RNA. According to the SPIDIA protocol, participants were instructed to store PAXgene Tube D at RT (PAX-RT) and EDTA tubes at 4°C or RT (EDTA-4°C and EDTA-RT, respectively), the distributions of gene expression analysis of FOS and IL8 with respect to these protocol conditions.

For both FOS and IL8, a statistically significant difference was observed depending upon the blood collection tube used (Fig. 3A, 3C) for both RNA C (FOS: Kruskal Wallis p-value <0.001; IL8: Kruskal Wallis p-value <0.001) and RNA D (FOS: Kruskal Wallis p-value = 0.003; IL8: Kruskal Wallis p-value <0.001). Moreover, we observed a relevant discrepancy between gene expression in RNA samples depending upon storage temperature and collection tube type (Fig. 3B, 3D). Specifically, we observed that IL8 levels (Fig. 3D) in RNA extracted from blood samples collected in EDTA tubes stored at RT before RNA extraction differed significantly from that in RNA from EDTA tubes stored at 4°C (Bonferroni adjusted p-value <0.001) and PAXgene tube (RT) (Bonferroni adjusted p-value <0.001). This difference was observed also for FOS (Fig. 3B) between RNA samples from EDTA tubes stored at 4°C and RNA from blood collected PAXgene tubes stored at RT (Bonferroni adjusted p-value <0.001).

## Discussion

One of the main purposes of the SPIDIA project was to investigate the role of pre-analytical variables in the handling of blood samples for molecular testing. In order to obtain evidence-based guidelines for the pre-analytical processing of blood samples for RNA analysis, two EQAs were planned (first and second SPIDIA-RNA). For each EQA, a survey and a proficiency testing program was implemented to evaluate current sample handling parameters and technologies for blood RNA-based analysis in laboratories in Europe. In addition to providing objective measurements for laboratory proficiency, we designed this EQA to approximate blood collection, specimen storage, and RNA extraction methods currently used by participating laboratories and compare the quality of the RNA produced by these methods.

Pursuant to these goals, we collected blood into EDTA-filled blood collection bags for this second EQA instead of in the CPDA blood bags used in the first EQA [1]. Even if this model does not follow the conventional procedure for blood collection for RNA analysis (as blood sample would be drawn directly into a tube rather than into a bag), the proposed procedure is able to provide a blood sample that mimics a real clinical blood sample and in a sufficient amount for the SPIDIA-RNA EQA set-up.

Because blood from one donor was not of sufficient volume to provide specimens for all of the participating laboratories, we determined the effect of blood pooling on gene expression. The results (Fig. 2) demonstrated that differential gene expression was observed between pooled and non-pooled blood for the IL1B transcript. Consequently, specimen pooling was abandoned as a proficiency specimen strategy in the second EQA. Blood from two donors were collected and aliquoted into proficiency specimens, and the participating laboratories were randomized into two groups, each group receiving blood specimens from only one donor.

Relative to the first SPIDIA-RNA EQA [1], other modifications were introduced including controlled shipping conditions (shipping containers with a temperature maintained at 2°C to 8°C for 48 h) and defined time and temperature storage conditions of proficiency specimens prior to RNA extraction (Fig. S2).

One hundred twenty-two applications were received from 21 different European countries, 109 laboratories returned the extracted RNA to the SPIDIA facility by the established deadline. During the first SPIDIA-RNA EQA, there were 124 applications, and 93 laboratories returned RNA samples to the SPIDIA facility [1]. The high response rate from the laboratories for both EQAs indicated a high level of interest and participation both in terms of the number of laboratories enrolled as well as the number of returned RNA samples (about 92% in both EQAs).

The survey queried current laboratory policies and practices specific to specimen handling. Respondents were asked to provide information on blood collection and extraction protocols (Table S1). The analysis of the survey from the second SPIDIA RNA-EQA confirmed the results obtained during the first EQA, which was the preference to use commercially available extraction kits (mainly silica membrane technology). The majority of the laboratories collected blood in K_2_EDTA tubes (66%), whereas others (21%) used PAXgene tubes. The quality of the extracted RNA samples was evaluated for yield and purity by UV analysis. Purified RNA was most often stored at −80°C, and the predominant downstream analytical methods were PCR technologies (qualitative and quantitative RT-PCR). Other aspects of sample handling and analysis protocols were more variable and included the volume of blood used and time and temperature of specimen storage post-phlebotomy.

Using the same approach adopted in the first SPIDIA EQA, we evaluated the quality of RNA returned to the SPIDIA facility by participating laboratories. An individual report for each laboratory was produced which described the proficiency of the laboratory respect to the results of the other participants. The distribution of the results, similar within each donor (see the report in Appendix S1), showed a median value of RNA purity (A_260_/A_280_) close to 2.0 indicating high quality RNA [15], [16] for RNA C as measured both by the participants and the SPIDIA laboratory. The same findings were observed for total RNA yield (ng/µL blood).

In addition, the distributions of the RIN scores were similar for the two donors. As no external reference value was adopted for the evaluation of laboratory proficiency, we classified as “in control” all RIN scores above the WL of 6.90 for Donor1 and 6.62 for Donor2. Only RIN scores ≥8.0 are classified as high integrity RNA [16]. In this study, the median RIN score was ≥8 for both donors (D1 median = 8.60; D2 median = 8.15) indicating high integrity of the extracted RNA for the majority of the returned RNA samples. The analysis of RT-qPCR kinetics revealed that only few samples showed the presence of RT-qPCR interferences.

Taken altogether, the analysis of these RNA quality parameters indicated that participants were proficient in the pre-analytical aspects of specimen handling for RNA analysis. The analysis of the gene expression in resultant RNA, however, demonstrated that pre-analytical factors, independent of the proficiency of the laboratory, significantly affected the quantity of some gene transcripts relative to T_0_ copy numbers. Whereas a narrow distribution of the expression levels four gene transcripts in all Tube C samples was observed (Appendix S1, Section C, C.1), a significant difference was evident in the expression levels of FOS and IL8 depending upon blood collection tube type (EDTA or PAXgene tubes) (Fig. 3A, 3C, respectively). The more homogeneous PAXgene sample gene expression distribution could be, partially, due to the use of the same extraction procedure specific for PAXgene Blood RNA tube (PAXgene Blood RNA kit).

From these results, we concluded that the presence of a stabilizer in the PAXgene tubes apparently maintained gene expression levels of FOS and IL8 close to those measured in T_0_ samples in RNA C (stored at RT for 24 h) and D (stored 48 h post-phlebotomy) (Fig. 3A and 3C). In contrast, the gene expression levels of FOS and IL8 in RNA isolated from EDTA blood collection tubes stored at either 4°C or RT showed an *ex vivo* gene-dependent induction 24 h after blood collection (Fig. 3A, 3C, Tube C). For IL8, this gene induction was more evident when blood was stored for 48 h after phlebotomy at RT without stabilizer (Fig. 3D). Similar results were obtained with gene expression analysis of the up- and down-regulated EDTA biomarkers FOSB and TNFRSF10c (Appendix S1, Section F).

An analysis of individual participant reports according to our proficiency classification scheme revealed that the distribution of the overall proficiency ratings was similar within the two donors with almost 40% of laboratories receiving “in control” assessment for all the considered quality parameters. This result was an overall increase in proficiency level in comparison to those obtained in the first SPIDIA-RNA EQA. This improvement was most likely due to the changes in the second EQA study design (proficiency specimen preparation, shipping) and pre-analytical specimen handling protocol (stringent time/temperature conditions).

In conclusion, the SPIDIA-RNA EQAs identified the most critical steps in the pre-analytical procedure concerning blood collection and processing for RNA testing. Furthermore, due to the improvements we adopted in the second EQA, we were able to make important conclusions regarding pre-analytical conditions, which affect *ex vivo* changes in the gene expression profile. These changes include gene induction, gene down-regulation, and RNA degradation, all of which could result in erroneous measurements of gene transcript levels [17]–[19]. Our results demonstrated that the use of PAX gene RNA Blood collection tube allows reliable gene expression analysis within 48 h from blood collection. Other blood collection tubes containing RNA stabilizers are commercially available but have not been tested in this study.

When using blood collection tubes which do not contain any RNA profile stabilizer (i.e. EDTA blood collection tubes, mostly used for cellular RNA analysis), it is strongly recommended to investigate whether a specific RNA species intended to be analyzed in the analytical test is stable after blood draw for the duration of the entire pre-analytical workflow.

The results of these two SPIDIA RNA EQAs studies have been proposed for use in the development of a Technical Specification by the European Committee for Standardization (CEN).

## Supporting Information

Figure S1 Distribution of participant laboratories (n = 124) through European Countries (A), Structures (B) and the main Research area (C).
(TIF)

Figure S2 Schematic comparison of the general workflow of the two SPIDIA-RNA EQAs: the first SPIDIA-RNA EQA study published by Pazzagli M et al. [1] (left side) and the second SPIDIA-RNA EQA reported in this publication (right side). In the two EQAs blood was drawn from different numbers of donors into blood bags containing different formulations of anticoagulant. In the second EQA blood from two donors was not pooled, blood aliquots intended to stay unstabilized were transferred into empty evacuated tubes (EST) instead of EDTA tubes and all tubes were shipped the same day of sample aliquoting to the participants under improved shipping conditions as indicated. EST = Evacuated Secondary Tube, that does not contain any chemical formulation. day 1, day 2, day 3 = Time period between blood collection and RNA preparation.
(TIF)

Table S1 Questionnaire: distribution frequencies. Usual procedures performed by participant laboratories (n = 92).
(DOC)

Appendix S1 Report for participant. Report, related to Donor1, produced for each participant containing the overall distribution of the analyzed RNA quality parameters and the specific evaluation of the performance for each parameter and overall evaluation.
(PDF)

Protocol S1 Protocol A- PAXgene Blood RNA tube. Procedures and protocol for blood storage and RNA extraction for participants receiving blood collected in PAXgene Blood RNA tubes.
(PDF)

Protocol S2 Protocol B- EDTA tubes (+4°C). Procedures and protocol for blood storage and RNA extraction for participants receiving blood collected in EDTA tubes, which had to store the blood at +4°C.
(PDF)

Protocol S3 Protocol B- EDTA tubes (RT). Procedures and protocol for blood storage and RNA extraction for participants receiving blood collected in EDTA tubes, which had to store the blood at RT.
(PDF)

Protocol S4 Result form – Protocol A - PAXgene Blood RNA tubes. Form to fill by experimental data performing RNA extraction from blood collected in PAXgene Blood RNA tubes.
(PDF)

Protocol S5 Result form – Protocol B - EDTA. Form to fill by experimental data performing RNA extraction form blood collected in EDTA tubes**.**
(PDF)
