# Supplementary figures and images for: SPIDIA-RNA: Second External Quality Assessment for the Pre-Analytical Phase of Blood Samples Used for RNA Based Analyses
Source: PLoS One. 2014 Nov 10;9(11):e112293. doi: 10.1371/journal.pone.0112293 (PMC4226503; doi:10.1371/journal.pone.0112293)

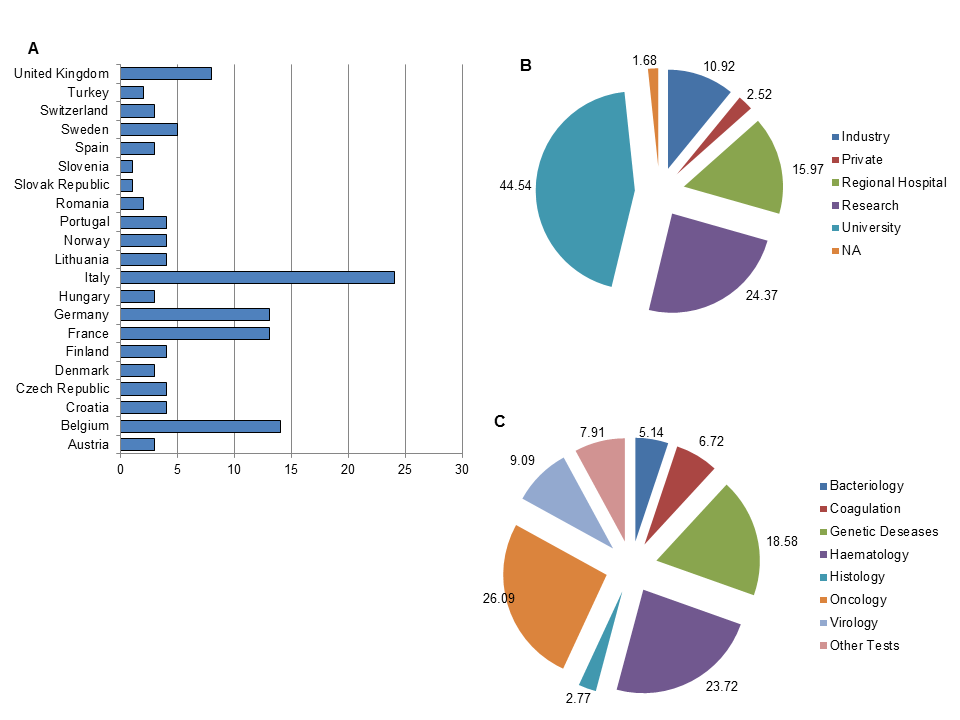

Supplement: Figure S1 — Distribution of participant laboratories (n = 124) through European Countries (A), Structures (B) and the main Research area (C). (TIF) [file pone.0112293.s001.tif]

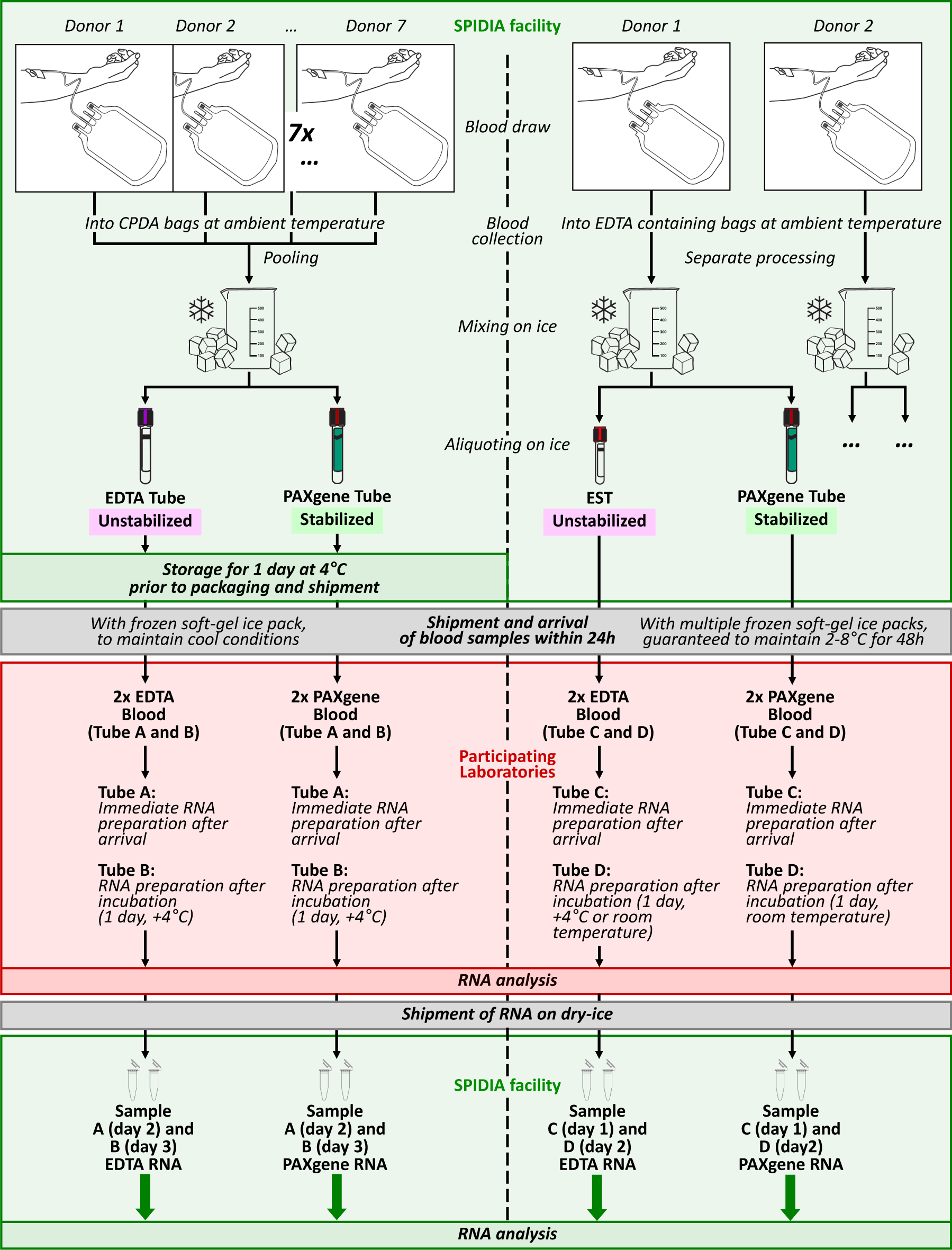

Supplement: Figure S2 — Schematic comparison of the general workflow of the two SPIDIA-RNA EQAs: the first SPIDIA-RNA EQA study published by Pazzagli M et al. [1] (left side) and the second SPIDIA-RNA EQA reported in this publication (right side). In the two EQAs blood was drawn from different numbers of donors into blood bags containing different formulations of anticoagulant. In the second EQA blood from two donors was not pooled, blood aliquots intended to stay unstabilized were transferred into empty evacuated tubes (EST) instead of EDTA tubes and all tubes were shipped the same day of sample aliquoting to the participants under improved shipping conditions as indicated. EST = Evacuated Secondary Tube, that does not contain any chemical formulation. day 1, day 2, day 3 = Time period between blood collection and RNA preparation. (TIF) [file pone.0112293.s002.tif]
