# Supplementary material for: SPIDIA-RNA: Second External Quality Assessment for the Pre-Analytical Phase of Blood Samples Used for RNA Based Analyses
Source: PLoS One. 2014 Nov 10;9(11):e112293. doi: 10.1371/journal.pone.0112293 (PMC4226503; doi:10.1371/journal.pone.0112293)
Supplement: Table S1 — Questionnaire: distribution frequencies. Usual procedures performed by participant laboratories (n = 92). (DOC) [file pone.0112293.s003.doc]

**Table S1 - Questionnaire** **distribution frequencies.**  Usual procedures performed by participant laboratories (n= 92).

| **Questions** |  | **N** | **%** |
| --- | --- | --- | --- |
| 1 - In which tube do you usually perform blood collection? | K_2_EDTA Tube  NaCitrate Tube  PAXgene blood RNA tube  Tempus Tube  Li-Heparine Tube  Other | 61  1  19  1  -  10 | 66  1  21  1  -  11 |
| 2 - Do you collect the blood on your own or do you receive the blood collected from elsewhere? | Collect  Receive  Collect and receive | 7  53  32 | 7  58  35 |
| 3 - How many milliliters of blood do you collect? | 1 mL ≤ mL ≤ 5 mL  5 mL ≤ mL ≤ 10 mL  >10 mL | 21  14  4 | 54  36  10 |
| 4- How many milliliters of blood do you receive? | 1 mL ≤ mL ≤ 5 mL  5 mL ≤ mL ≤ 10 mL  >10 ml | 42  32  11 | 49  38  13 |
| 5 - How long is the usual time interval between the blood collection/receipt and the RNA extraction? | ≤ 12 h  12 h < h ≤ 24 h  24 h < h ≤ 480 h  >480 h | 39  29  15  9 | 42  32  16  10 |
| 6- Only for those that receive blood from elsewhere: at what temperature is the collected blood usually delivered to you? | -80°C  -20°C  4°C  RT | 3  4  25  53 | 4  5  29  62 |
| 7 - At what temperature you store the collected blood before RNA extraction? | -80°C  -20°C  4°C  RT  missing | 10  12  55  14  1 | 11  13  60  15  1 |
| 8 - What is the procedure for RNA extraction?   1. Do you use a kit? 2. If yes, what is the kit? 3. The procedure is: | Yes  No  Magnetic Bead  PAXgene  Precipitation  Silica  Missing  Automatic  Manual  missing | 79  13  5  19  5  41  9  22  69  1 | 86  14  6  25  6  52  11  24  75  1 |
| 9 - How many milliliters of blood do you use for RNA extraction? | ≤ 5 mL  >5 mL  missing | 66  22  4 | 72  24  4 |
| 11 - Do you evaluate the yield and purity of extracted RNA?  If the answer is "Yes": what is the method? | Yes  No  Agilent Technology  RiboGreen  Spectrophotometer  Agilent technology + Spectrophotometer  Other | 80  12  1  1  74  2  2 | 87  13  1  1  92  3  3 |
| 12 - How long is the usual time interval between the RNA extraction and concentration evaluation? | ≤ 6 h  6 h < h ≤ 24 h  >24h  missing | 79  5  4  4 | 86  6  4  4 |
| 13 - What kind of analysis do you usually perform on your extracted RNA? | Microarray evaluation  RIN evaluation  RT and PCR  RT and qPCR  RIN + RT and qPCR  RIN + RT and PCR + RT and qPCR  RT and qPCR +microarray evaluation  RT and PCR + RT and qPCR + microarray  RT and PCR + RT and qPCR | 1  3  9  64  2  2  1  1  3 | 1  4  11  74  2  2  1  1  4 |
| 14- How long is the time interval between the RNA extraction and analysis of RNA? | ≤ 6 h  6 h < h ≤ 24 h  24 h < h ≤ 120 h  120 h < h ≤ 240 h  >240 h  missing | 21  34  22  3  11  1 | 23  37  24  3  12  1 |
| 15- At what temperature do you usually store the extracted RNA? | -80°C  -20°C | 74  18 | 80  20 |
| 16- For how long do you usually store RNA? | No storage  Days  Months  Years | 2  4  12  74 | 2  5  13  80 |
| 17- Is your laboratory accredited to perform molecular diagnostic tests?  If yes, which kind of accreditation do you have? | Yes  No  missing  Academic  ISO 15189  Institutional  Regional  Other | 49  42  1  5  22  7  3  12 | 53  46  1  10  45  14  6  25 |
