# Supplementary material for: SPIDIA-RNA: Second External Quality Assessment for the Pre-Analytical Phase of Blood Samples Used for RNA Based Analyses
Source: PLoS One. 2014 Nov 10;9(11):e112293. doi: 10.1371/journal.pone.0112293 (PMC4226503; doi:10.1371/journal.pone.0112293)
Supplement: Appendix S1 — Report for participant. Report, related to Donor1, produced for each participant containing the overall distribution of the analyzed RNA quality parameters and the specific evaluation of the performance for each parameter and overall evaluation. (PDF) [file pone.0112293.s004.pdf]

SPIDIA – 2nd RNA ring trial: Laboratory report

Lab ID: XXX – Donor 1

A. Purity and Quantity of RNA C

A.1. Spectrophotometric data provided by your Lab and by Spidia

260nm	280nm	320nm	Purity Lab	Quantity Lab (ng/µl blood)	Purity Spidia	Quantity Spidia (ng/µl blood)	Dilution factor	Extraction vol. (µl)	Elution vol. (µl)	Buffer	DNase treatment
0.375	0.265	0.012	1.42	30.00	1.79	22.08	10	250	50	Nuclease Free Water	No

A.2. Additional information provided by your Lab

Time interval (hours)		Temperature of RNA storage (°C)		Extraction method		Spectrophotometer	
arrival to extraction	extraction to analysis	arrival to extraction	extraction to analysis	producer	supplier	producer	supplier
4.25	960.00	4.0	-20.0	TRIzol Reagent - Invitrogen	15596-026	Eppendorf BioPhotometer	6131 000.012

A.3. Your Lab (●) versus overall distribution – Purity

In the figures the blue lines represent the Action Limits (ALs) and the gray lines represent the Warning Limits (WLs).

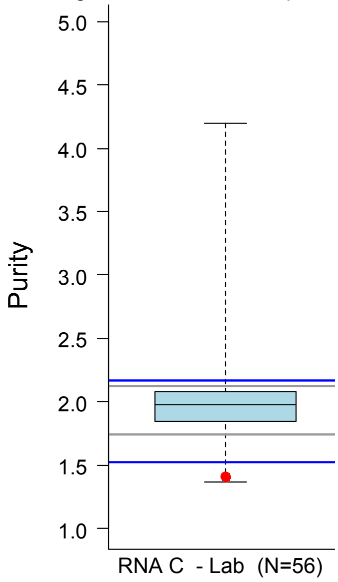

Lower AL=1.53; Upper AL=2.16
Lower WL=1.74; Upper WL=2.12

out of control

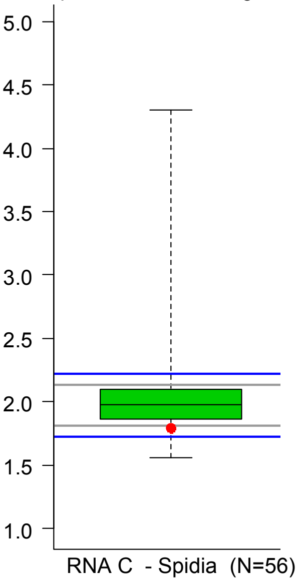

Lower AL=1.73; Upper AL=2.22
Lower WL=1.81; Upper WL=2.13

warning

A.4. Your Lab (●) versus overall distribution – Quantity

In the figures the blue line represents the Action Limit (AL) and the gray line represents the Warning Limit (WL).

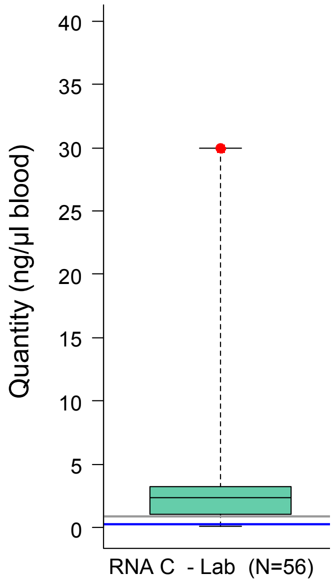

AL= 0.26; WL= 0.86

in control

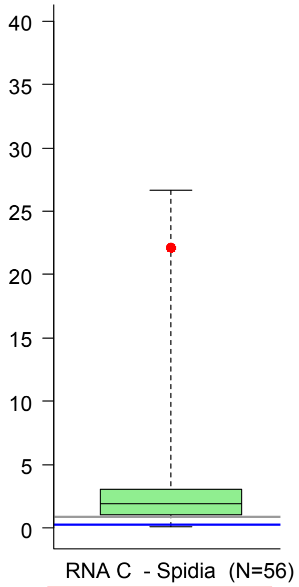

AL=0.31; WL=0.89

in control

Lab ID: XXX – Donor 1

B. Integrity of RNA C

B.1. Your Lab (●) versus overall distribution – RIN

In the figure the blue line represents the Action Limit (AL) and the gray line represents the Warning Limit (WL).

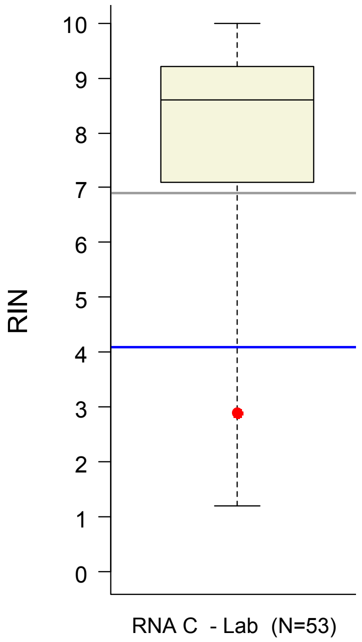

AL= 4.09; WL= 6.90

out of control

B.2 Electropherogram Agilent – RNA C

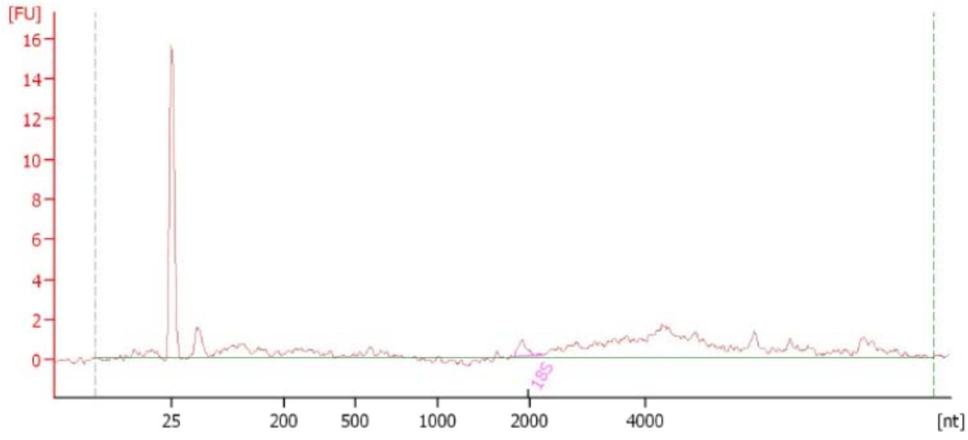

Lab ID: XXX – Donor 1

C. Quantification of four genes by real-time PCR on RNA C

C.1. Your Lab (●) versus overall distribution

In the figures the blue lines represent the Action Limits (ALs) and the gray lines represents the Warning Limits (WLs).

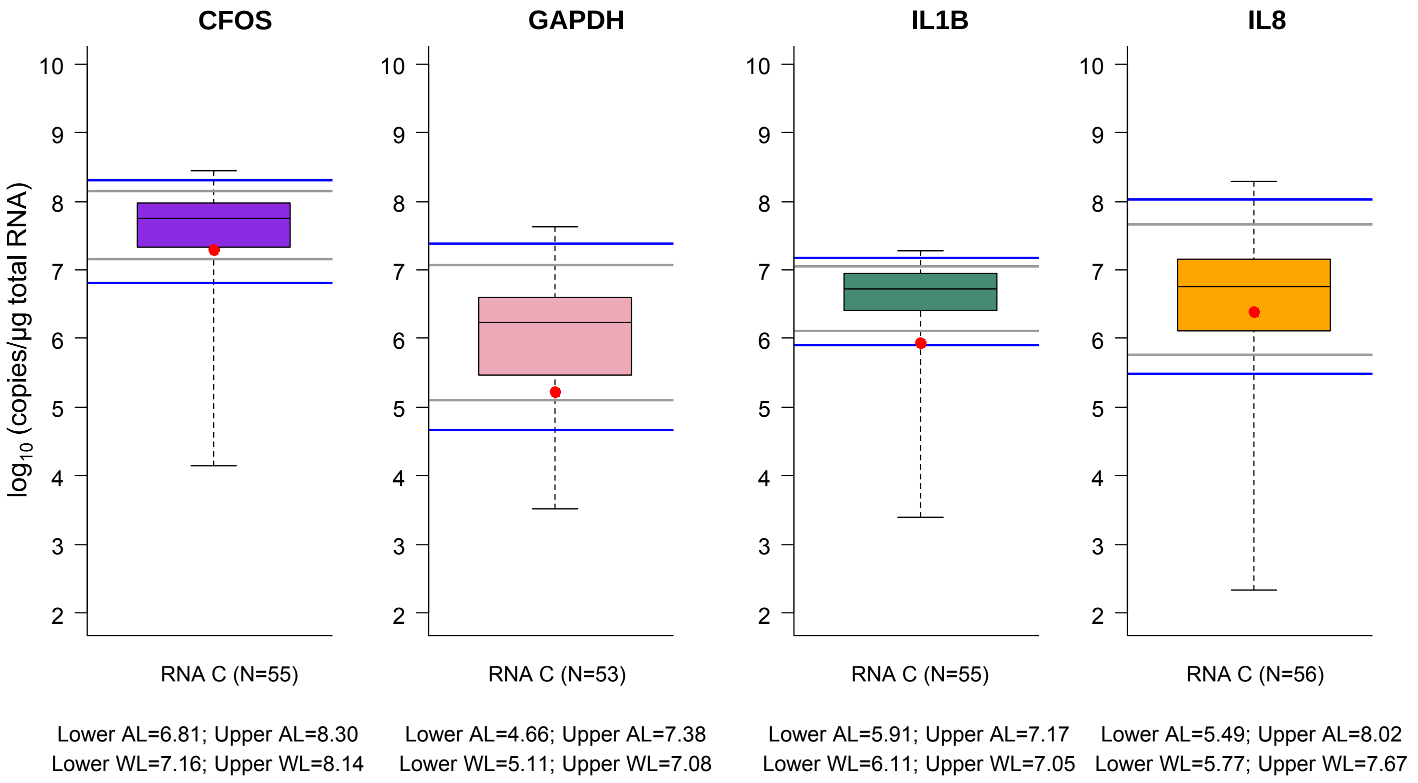

CFOS	GAPDH	IL1B	IL8
in control	*in control*	*warning*	*in control*

Lab ID: XXX – Donor 1

D. Interferences in quantification of four genes by real-time PCR on RNA C

D.1. Your Lab (●) versus overall distribution

In the figures the two lines represent the two Kineret thresholds for outliers identification: 5.99 (weak outlier) and 9.21 (strong outlier).

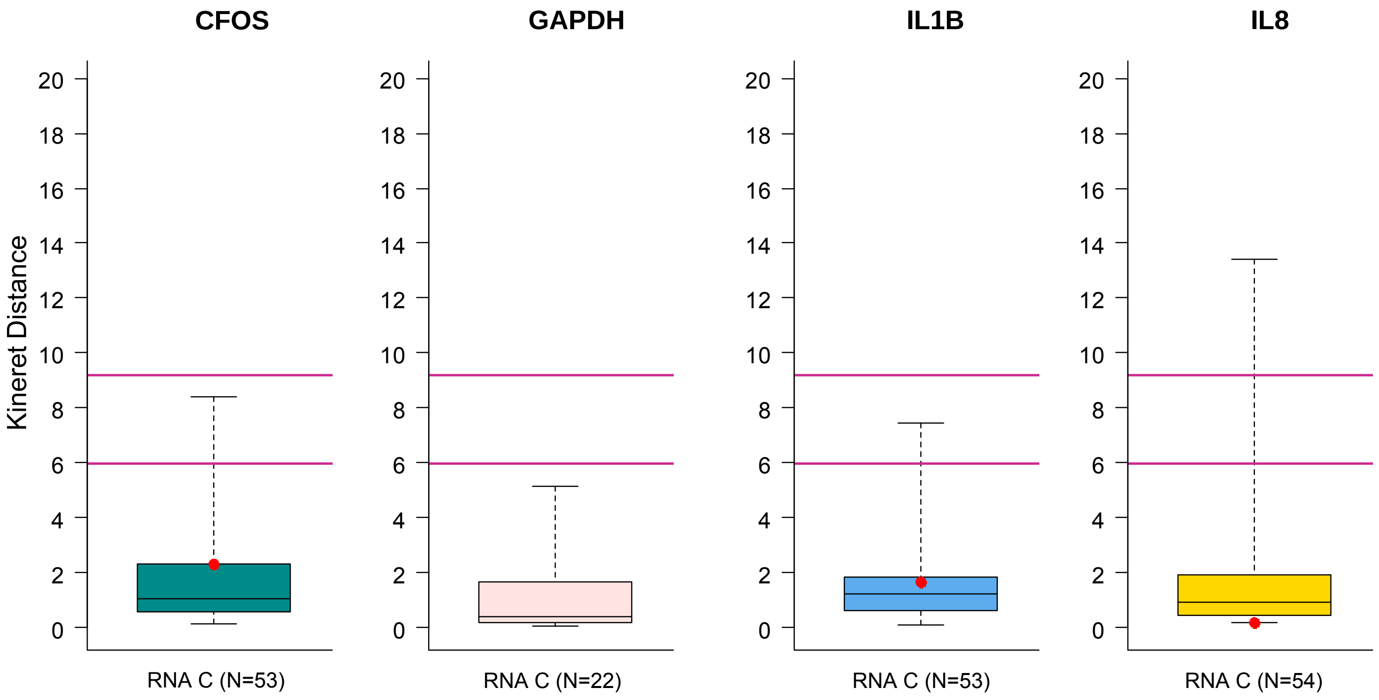

CFOS	GAPDH	IL1B	IL8
in control	*missing*	*in control*	*in control*

Lab ID: XXX – Donor 1

E. Summary

	Performance			Missing	Comments
A.3. RNA C – Purity Lab			out of control		
A.4. RNA C – Quantity Lab	in control				
A.3. RNA C – Purity Spidia		warning			
A.4. RNA C – Quantity Spidia	in control				
B.1. RNA C – Integrity			out of control		
C.1. RNA C – CFOS qPCR	in control				
C.1. RNA C – GAPDH qPCR	in control				
C.1. RNA C – IL1B qPCR		warning			
C.1. RNA C – IL8 qPCR	in control				
D.1. RNA C – CFOS interferences	in control				
D.1. RNA C – GAPDH interferences				missing	The Kineret analysis did not provide any result
D.1. RNA C – IL1B interferences	in control				
D.1. RNA C – IL8 interferences	in control				

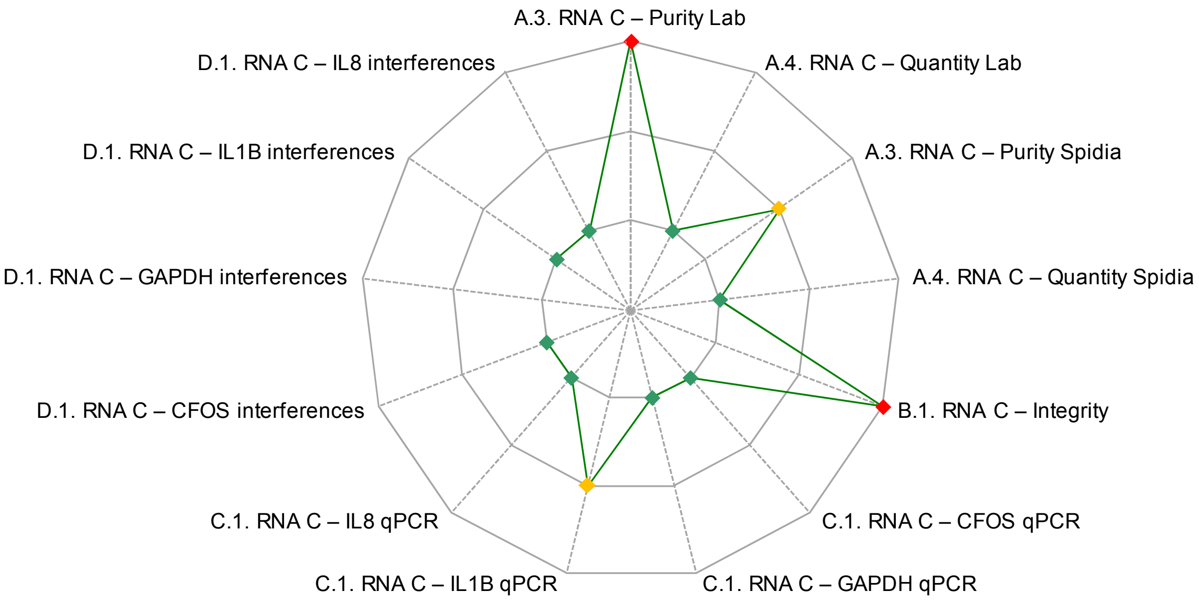

Lab ID: XXX – Donor 1

F. Appendix

F.1. Relative quantification of the EDTA up-regulated biomarker by real-time PCR

In the figures the horizontal blue line is plotted in correspondence of log_2 (RQ) = 0

Tube C

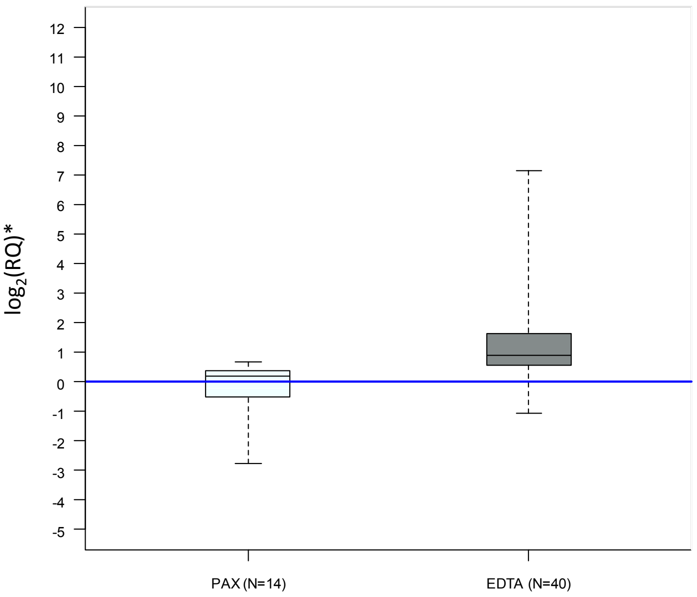

Tube D

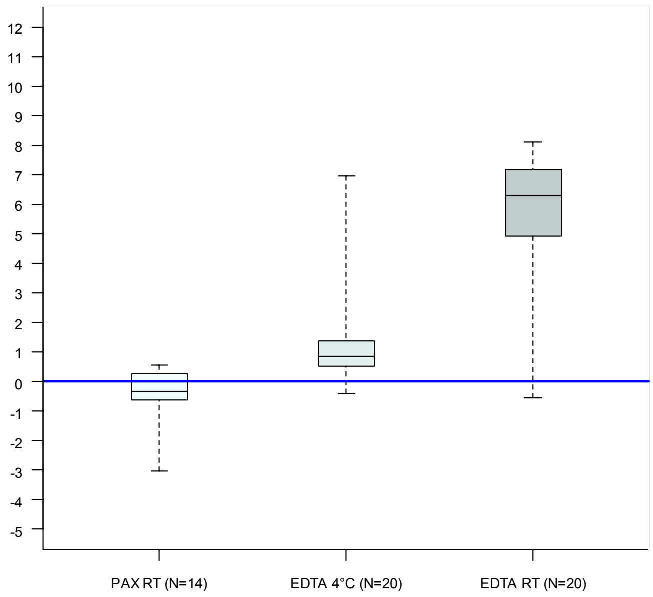

* RQ= $2^{-\Delta\Delta Ct}$

F.2. Relative quantification of the EDTA down-regulated biomarker by real-time PCR

In the figures the horizontal blue line is plotted in correspondence of log_2 (RQ) = 0

Tube C

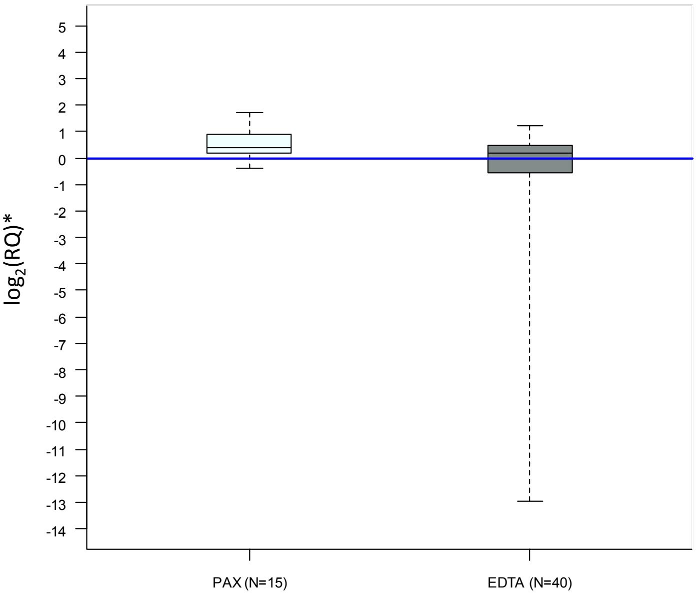

Tube D

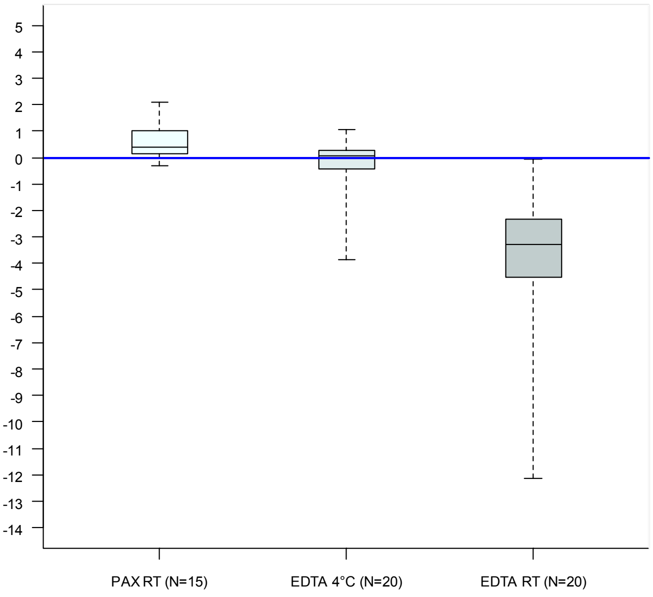

* RQ= $2^{-\Delta\Delta Ct}$

This report has been produced with the collaboration of the **University of Florence** (M.Pazzagli, S.Gelmini, C.Orlando, L.Simi, F.Malentacchi), **Fondazione IRCCS Istituto Nazionale dei Tumori of Milan** (P.Verderio, S.Pizzamiglio, C.Ciniselli), **QIAGEN** (R.Wyrich, K.Günther, C.Hartmann, H.Ibrahim), **TATAA BIOCENTER** (A.Tichopad) **LABONNET** (T.Bar).
