# Supplementary material for: SPIDIA-RNA: Second External Quality Assessment for the Pre-Analytical Phase of Blood Samples Used for RNA Based Analyses
Source: PLoS One. 2014 Nov 10;9(11):e112293. doi: 10.1371/journal.pone.0112293 (PMC4226503; doi:10.1371/journal.pone.0112293)
Supplement: Protocol S3 — Protocol B- EDTA tubes (RT). Procedures and protocol for blood storage and RNA extraction for participants receiving blood collected in EDTA tubes, which had to store the blood at RT. (PDF) [file pone.0112293.s007.pdf]

SCHEME OF PROCEDURE:
SPIDIA-RNA
pre-analytical survey
Protocol B – **EDTA** tubes (RT)
2nd ring trial

Dear participant,

SPIDIA (www.spidia.eu) thanks you to join us to this pre-analytical external quality assessment survey for RNA. SPIDIA will contribute to the standardisation and improvement of procedures and tools for pre-analytical intervention. The individual steps, such as sample handling, stabilisation and storage, will be standardised and integrated in one holistic process combining classical and molecular diagnostics. SPIDIA aims at developing and validating the necessary guidelines and tools that will make possible the production of new knowledge and its translation into practical applications in the area of health and medicine.

The aim of SPIDIA-RNA is to evaluate the stability and integrity on RNA from blood sample, in particular focusing the analysis in relation of :

- the method of sample collection,
- the time interval between collection and extraction,
- the procedure of extraction,
- the storage and shipping of extracted sample.

In this call, SPIDIA asks you to perform RNA extraction from two blood samples collected in EDTA tubes sent by SPIDIA.

Please, find here enclosed all the details necessary to perform this SPIDIA-RNA survey.

Good luck and our best regards,

Uwe Oelmueller
Coordinator of the SPIDIA Project

Mario Pazzagli
Leader of the WP 1.2: Evidence-based Quality Guidelines for the pre-analytical phase of Blood Samples

TO BE PERFORMED IMMEDIATELY UPON ARRIVAL:

- DO NOT DISCARD THE SPIDIA BOX SINCE IT IS NECESSARY FOR THE SHIPMENT OF YOUR SAMPLES.
- CHECK THE AVAILABILITY OF DRY ICE (3 kg) IN YOUR LABORATORY SINCE IT WILL BE NECESSARY TO PERFORM THE SHIPMENT OF YOUR SAMPLES TO SPIDIA UNFI LABORATORY.

SUMMARY OF THE SCHEME:

- Why have I received two samples?
- What does the shipping box contain?
- What do you do with the samples?
- How do you send your results to SPIDIA?
- How do you send your samples to SPIDIA?

Why have I received two samples?

You receive two blood samples in EST tubes: Tube C XXX and Tube D XXX (XXX is our internal number referring to your lab).
Please, extract both of them by using your procedures and following this protocol.

You will receive the report of your performance only about RNA extracted from Tube C;
Tube D will be used for evidence based guidelines, you will be not judged on this result.

What does the shipping box contain?

In this box you find the samples, the protocol and the Result Form to record your data.

- 1 vial labelled Tube C XXX contains 3 ml of whole blood in a EST tube. Blood was collected using EDTA as anticoagulant.
- 1 vial labelled Tube D XXX contains 3 ml of whole blood in a EST tube. Blood was collected using EDTA as anticoagulant.
- 2 empty vials labelled CXXX and DXXX to send back the extracted RNAs (RNA C and RNA D, respectively) to SPIDIA UNIFI laboratory.
- 1 empty vial labelled EBXXX to send back the buffer used for RNAs elution/suspension.
- 1 label with SPIDIA UNIFI laboratory address:

SPIDIA UNIFI laboratory:
Prof. Mario Pazzagli
Dept. Clinical Physiopathology
O.U. Clinical Biochemistry
Viale G. Pieraccini, 6
50139 FLORENCE-ITALY

* please do not discard the shipping box (polystyrene box). You will use it to send back your RNA samples with DRY ICE (on your charge) to the SPIDIA UNIFI laboratory.

What do you do with the samples?
Before starting the extraction, please read the result form and record all necessary information.
Blood Sample
Please, remind to invert the tubes several times before the extraction, then spin down briefly (1-3 seconds) just to remove blood from the cap, be careful not to spin down the blood cells, since this will reduce the RNA yield.

- Take Tube D XXX and put it at Room Temperature (RT)for 24h hours (it must be extracted 24h after tube C).

- Take Tube C XXX and equilibrate it to room temperature before to extract RNA. Perform extraction of RNA immediately (see **Note 1**) following your own procedures.

- After RNA extraction from Tube C XXX, transfer the eluate to the vial labelled CXXX (RNA C), immediately perform the spectrophotometric measurements (see **Note 2**) on the extracted RNA (see Spectrophotometric evaluation procedure).

- Store CXXX at -20°C or -80°C until the shipping to SPIDIA UNIFI laboratory.

- 24h after Tube C RNA extraction, take Tube D XXX and extract corresponding RNA, following your own procedures.

- After RNA extraction from Tube D XXX, transfer the eluate to the vial labelled DXXX (RNA D), immediately (see **Note 3**) perform the spectrophotometric measurements on the extracted RNA (see Spectrophotometric evaluation procedure).

- Store DXXX at -20°C or -80°C until the shipping to SPIDIA UNIFI laboratory.

-Transfer 30µl of RNA elution/resuspension buffer in the EBXXX labelled vial and store it at room temperature. Please use the same buffer to elute/resuspend RNA C and RNA D.

Note 1. In order to minimize the impact of shipping and storage on the results, it is essential to extract Tube C immediately after arrival. If it is not possible to extract immediately the RNA from blood, store Tube C at 4°C, and extract RNA as soon as possible. If Tube C is not extracted immediately, it has to be stored at 4°C until extraction and store also Tube D at 4°C until you start the extraction from Tube C. Put Tube D at room temperature when you start extraction of Tube C and incubate Tube D for 24 h at room temperature, as described above (**remember that, in any case, Tube D MUST be extracted 24h after Tube C, after a 24h incubation at RT**).
Note 2. If it is not possible to perform spectrophotometric measurements immediately after extraction on RNA C, we suggest to store RNA C at -20°C or -80°C in the meantime.
Note 3. If it is not possible to perform spectrophotometric measurements immediately after extraction on RNA D, we suggest to store RNA D at -20°C or -80°C in the meantime.

Spectrophotometric evaluation procedure
By spectrophotometric measurements you evaluate RNA concentration (ng/µl; C = 260nm x 40 x dilution factor or C = (260nm-320nm) x 40 x dilution factor) and purity (R = 260nm / 280nm or R=(260nm-320nm) / (280nm-320nm)) in the two extracted RNAs (CXXX and DXXX) from blood samples. Record all the data (Absorbance at 320nm, 260nm, 280nm, buffer used for dilution, dilution factor) in the Result Form.
During the spectrophotometric measurements calibrate correctly your UV blank:

- if you measure a diluted sample: use the same solution (water or buffer) that has been used for RNA dilution;
-if you measure an entire sample: use the same solution (water or buffer) that has been used for RNA elution/resuspension

Specify in the Result form which type of blank you used.

How do you send your results to SPIDIA?

Data collection: Result Form

To minimise any error in the data collection we provide you a double procedure: Result Form has to be completed by using both the paper copy and the on-line version. We encourage to store a copy of your Result Form for any further requirement.

Please, complete both the on-line and the paper version of the Result Form

Before the shipment, enclose a copy of the paper version in the shipment box together with the extracted RNAs.

To enter in the personalised area of the SPIDIA website:

1. connect to the SPIDIA web site http://www.efcclm.eu/spidia/index.htm
2. select "Participant" from the "login area" section
3. upload your page using your login and password and record your data

If you do not have anymore the login data, please send a request to spidiaunifi@unifi.it

Data collection: Questionnaire

In the personalised area of SPIDIA website, fill the Questionnaire form. Please enclose a copy of the completed Questionnaire also in the shipment of the SPIDIA box.
At your request, to spidiaunifi@unifi.it, is also possible to receive, fill and send this form by e-mail.

How do you send your samples to SPIDIA?

Provide by yourself the dry ice. The dry ice is NOT provided by SPIDIA. The amount of dry ice must be about 3 kg to ensure the correct temperature during the shipping.

- Prepare the shipping box using the polystyrene box (the same in which you received the blood).
- Add the dry ice.
- Put the following material in the shipping box:
  - RNA C : CXXX
  - RNA D : DXXX
  - Elution/resuspension buffer: EBXXX
  - Filled Result Form
  - Filled the Questionnaire

Apply the enclosed label with SPIDIA UNIFI laboratory address to the shipping box.

Send it to SPIDIA UNIFI laboratory by phone calling DHL and selecting the correct code to perform the shipment:

- if your lab is extra-ITALY the code is : 951459463
- if your lab is in ITALY the code is: 105615282

The airway bill have to be requested to your local DHL.
Fill the DHL airway bill exactly as reported in the enclosed copy.

Shipment will be free of charge.

For any question you can contact us at: spidiaunifi@unifi.it
