# Supplementary material for: SPIDIA-RNA: Second External Quality Assessment for the Pre-Analytical Phase of Blood Samples Used for RNA Based Analyses
Source: PLoS One. 2014 Nov 10;9(11):e112293. doi: 10.1371/journal.pone.0112293 (PMC4226503; doi:10.1371/journal.pone.0112293)
Supplement: Protocol S4 — Result form – Protocol A - PAXgene Blood RNA tubes. Form to fill by experimental data performing RNA extraction from blood collected in PAXgene Blood RNA tubes. (PDF) [file pone.0112293.s008.pdf]

RESULT FORM

SPIDIA-RNA - Protocol A – PAXgene blood RNA tubes

Please, remind that Result Form have to be completed both in the on-line version and in the paper copy.

Attention: Please, fill out the form completely! You won't be able to change parts, selections or answers after sending.

Please insert the internal number referring to your lab (XXX) |_|_|_|

1. Status of Blood Samples

1.1 How is the status of the received blood samples?

Indicate the condition of the blood samples after arrival

**Is the blood coagulated?**	○ Yes	○ No
note		
**Is the blood hemolysed?**	○ Yes	○ No
note		

1.2 What is the temperature of the samples at the arrival?

Indicate the temperature of the blood samples at the arrival

Samples temperature		
When you open the package, the samples temperature is:	☐ cool	☐ RT
note		

2. Storage Time and Temperature of Blood Samples

Indicate the date and time of: blood samples arrival, date of RNA extraction and the blood samples storage temperature between blood samples arrival and RNAs extraction.

	Date and Time of Samples arrival		Date and Time of RNAs Extraction		Temperature of blood storage between arrival to RNA extraction
	(dd/mm/yy)	(hour:min)	(dd/mm/yy)	(hour:min)	(°C)
Tube C		:		:	
Tube D				:	

	Notes
Tube C	
Tube D	

3. Do you use DNAse treatment?

Do you use DNAse?	Yes	No

4. Storage Time and Temperature of extracted RNAs

Indicate the date and time of the spectrophotometer measurements and the storage temperature between RNAs extraction and spectrophotometric analysis.

	Date and Time of RNA spectrophotometric Analysis		Temperature of RNA storage between extraction to analysis
	(dd/mm/yy)	(hour:min)	(°C)
RNA C		:	
RNA D		:	

5. Volumes

Indicate the volume of blood used for RNAs extraction, the volume used to elute/resuspend extracted RNAs, the buffer used to elute/resuspend the RNAs

	Blood extraction volume (µl) (Volume of blood used for RNA extraction (µl))	Elution/Resuspension volume (µl) (Volume used for RNA elution/resuspension (µl))	Elution Buffer Used (Specify the name of solution used)
RNA C			
RNA D			

How to fill this table i.e.: you use all the blood PAXgene blood RNA content (2500µl) to perform the RNA extraction and you elute the RNA in 80µl of BR5 buffer:

	Blood extraction volume (µl) (Volume of blood used for RNA extraction (µl))	Elution/Resuspension volume (µl) (Volume used for RNA elution/resuspension (µl))	Elution Buffer Used (Specify the name of solution used)
RNA C	2500	80	BR5
RNA D	2500	80	BR5

6. Spectrophotometric Analysis

6.1 Spectrophotometer

	Producer/Supplier/Homebrew	Catalog Number/Model
Spectrophotometer		

6.2 Spectrophotometric Data

Please record the spectrophotometric data as follows by entering values with at least 3 decimal places. Insert the absorbance value you have obtained by measuring your RNA sample and in the case you measure a diluted RNA, the dilution factor you perform for analysis.

NOTE: Use “dot” as decimal separator

	A260	A280	A320*	Dilution factor**
RNA C				
RNA D				

*NOTE: If your Spectrophotometer is not equipped to read at 320nm put the value NA for A320
**If you don’t dilute the sample insert “1” as dilution factor

How to fill this table Example of spectrophotometric results if you evaluate 2µl of RNA sample diluted in 100µl of buffer:

	A260	A280	A320*	Dilution factor
RNA C	1.914	0.855	0.010	50

6.3 Please record RNA quantity (C) and purity (R) in the following table

Please note that in this Table quantity must be reported as concentration ng/µl (C=260nm x 40 x dilution factor or C=(260nm-320nm) x 40 x dilution factor) and purity by evaluating the ratio of absorbance value, R = 260nm/280nm or R=(260nm-320nm)/(280nm-320nm). Please record the data by entering values with at least 3 decimal places

NOTE: Use “dot” as decimal separator

	RNA quantity (C) (ng/µl)	RNA purity (R)	Notes
RNA C			
RNA D			

7. Shipping Time and Storage Temperature of RNAs

Record the date and the time you ship the samples to SPIDIA UNFI laboratory and the storage temperature of RNAs before to send them.

	Temperature of RNA storage before shipment (°C)
RNA C	
RNA D	

Date and Time of pick up delivery service at your lab	(dd/mm/yy)	(hour:min)
		:

Keep this completed Result Form for your record

Please check your data **carefully** - you will not be able to edit them later
